# Polygenic risk score predicts all-cause death in East Asian patients with prior coronary artery disease

**DOI:** 10.3389/fcvm.2024.1296415

**Published:** 2024-02-13

**Authors:** Min Qin, Yonglin Wu, Xianhong Fang, Cuiping Pan, Shilong Zhong

**Affiliations:** ^1^ School of Medicine, South China University of Technology, Guangzhou, China; ^2^ Department of Pharmacy, Guangdong Provincial People’s Hospital (Guangdong Academy of Medical Sciences), Southern Medical University, Guangzhou, China; ^3^ Center for Intelligent Medicine Research, Greater Bay Area Institute of Precision Medicine (Guangzhou), School of Life Sciences, Fudan University, Guangzhou, China; ^4^ Department of Cardiology, Guangdong Cardiovascular Institute, Guangdong Provincial People’s Hospital (Guangdong Academy of Medical Sciences), Guangzhou, China; ^5^ Center for Evolutionary Biology, Fudan University, Shanghai, China; ^6^ Guangdong Provincial Key Laboratory of Coronary Heart Disease Prevention, Guangdong Cardiovascular Institute, Guangdong Provincial People’s Hospital (Guangdong Academy of Medical Sciences), Guangzhou, China

**Keywords:** coronary artery disease, polygenic risk scores, all-cause death, prognostic model, secondary prevention

## Abstract

**Introduction:**

Coronary artery disease (CAD) is a highly heritable and multifactorial disease. Numerous genome-wide association studies (GWAS) facilitated the construction of polygenic risk scores (PRS) for predicting future incidence of CAD, however, exclusively in European populations. Furthermore, identifying CAD patients with elevated risks of all-cause death presents a critical challenge in secondary prevention, which will contribute largely to reducing the burden for public healthcare.

**Methods:**

We recruited a cohort of 1,776 Chinese CAD patients and performed medical follow-up for up to 11 years. A pruning and thresholding method was used to calculate PRS of CAD and its 14 risk factors. Their correlations with all-cause death were computed via Cox regression.

**Results and discussion:**

We found that the PRS for CAD and its seven risk factors, namely myocardial infarction, ischemic stroke, angina, heart failure, low-density lipoprotein cholesterol, total cholesterol and C-reaction protein, were significantly associated with death (*P* ≤ 0.05), whereas the PRS of body mass index displayed moderate association (*P* < 0.1). Elastic-net Cox regression with 5-fold cross-validation was used to integrate these nine PRS models into a meta score, metaPRS, which performed well in stratifying patients at different risks for death (*P* < 0.0001). Combining metaPRS with clinical risk factors further increased the discerning power and a 4% increase in sensitivity. The metaPRS generated from the genetic susceptibility to CAD and its risk factors can well stratify CAD patients by their risks of death. Integrating metaPRS and clinical risk factors may contribute to identifying patients at higher risk of poor prognosis.

## Introduction

Coronary artery disease (CAD) is the leading cause of death worldwide and associated with 17.8 million deaths annually (1–4). The number of people suffering from CAD has increased substantially over the past three decades. There are more than 197 million people living with CAD globally (5), resulting in huge expenditures on medical treatment, as well as secondary and tertiary preventions. Therefore, early identification of CAD patients at elevated risk of death permits precise delivery of more intense medical interventions and effective lifestyle changes, which are essential to decreasing mortality and reducing healthcare burden.

CAD is highly heritable with heritability estimated at 40%–60% (6). As such, genetic susceptibility profiles may confer the potential for early risk screening. During the last two decades, numerous genome-wide association studies (GWAS) were devoted to identifying single nucleotide variants (SNVs) associated with CAD and its risk factors by aggregating millions of samples (7–10). As germline DNA variation is quantifiable at the time of birth, which is before the onset of the disease or symptoms, and is therefore attractive for predicting the incidence of various cardiovascular diseases, including CAD (11), type 2 diabetes (12), atrial fibrillation (13), and myocardial infarction (14). Polygenic risk score (PRS), which integrates inherited risk inferred by GWAS into a single quantitative metric, has displayed its potential in various cardiovascular diseases to classify patients by risks of future incidence (15–17). Due to the broad availability of the GWAS data, PRS is readily applicable to many diseases. However, most studies thus far mainly focused on future incidence, i.e., primary prevention, in individuals of European ancestry without pre-existing CAD (15, 16, 18)^,^. Only a few studies explored whether the genetic susceptibility to CAD and its risk factors could predict risks of adverse outcomes in patients with pre-existing CAD (19, 20). Few examined that in patients of East Asian ancestry.

To address these needs, we recruited a prospective cohort consisting of 1,776 Chinese patients with pre-existing CAD and carried out medical follow-ups for up to 11 years. We aimed to leverage GWAS summary statistics derived from large cohorts of East Asians, such as Biobank Japan (9, 10), and develop the PRS models in our cohort for learning and adaptation. Our goal was to derive a meta PRS model for CAD patients of East Asian ancestry, which combined genetic profiles of CAD and its risk factors and was capable to predict the risk of poor prognosis.

## Methods

### Study population enrollment and baseline characteristics collection

This study was approved by the Medical Research Ethics Committee of Guangdong Provincial People's Hospital and complied with the Declaration of Helsinki. All patients provided written informed content.

**Figure 1:**
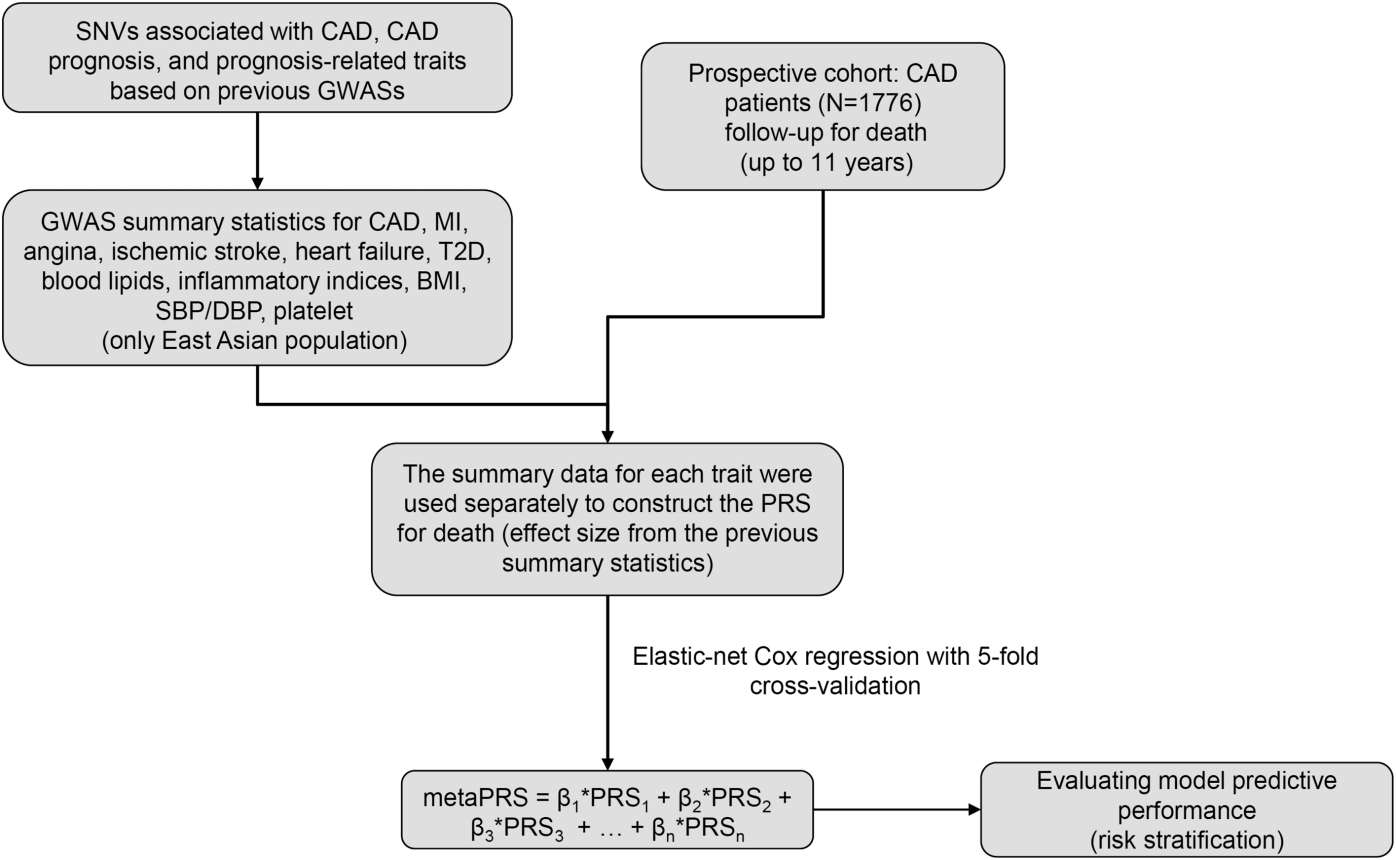
The overall design of this study. GWAS, genome-wide association study; CAD, coronary artery disease; MI, myocardial infarction; T2D, type 2 diabetes; BMI, body mass index; SBP, systolic blood pressure; DBP, diastolic blood pressure.

The overall design of this study was depicted in Figure 1. We recruited 1,776 patients diagnosed with CAD from Guangdong Provincial People's Hospital between January 2010 and December 2013 to form a prospective cohort. All individuals had stenosis of ≥50% in at least 1 major coronary artery by coronary angiography and/or a diagnosis of CAD based on the World Health Organization Criteria (21). Meanwhile, we obtained baseline characteristics, including demographics, medical history, biochemical measurements, and medication, from the hospital information database. In this study, subjects who met any of the following criteria were excluded: (1) renal dysfunction (defined as serum creatinine concentration >2 times the upper limit of normal (ULN) (230 µmol/L), or with history of renal transplantation or dialysis); (2) hepatic dysfunction [defined as serum transaminase concentration >2 times the ULN (80 U/L), or a diagnosis of cirrhosis]; (3) being pregnant or lactating; (4) diagnosed with advanced cancer or receiving hemodialysis; (5) history of thyroid disease and related medication; (6) blood sample and coronary angiography image unavailable; (7) missing follow-up information.

The endpoint of this study was an all-cause death event occurred during the follow-up period. All participants were followed prospectively and continuously by telephone contact with patients or their families every six months to collect the study's endpoint. The average follow-up time was 5 years and the maximum follow-up time was approximately 11 years.

### Single nucleotide variants genotyping and imputation

DNA was extracted on the hemocytes samples by TGuideM16 automatic nucleic acid extractor (Cat.NO. OSE-M16) with a matching extraction kit (Cat.NO. OSR-M102) of TIANGEN. Each individual was genotyped by the Global Screening Array (GSA) bead chip of Illumina, according to the standard protocol for Illumina Infinium HTS Assay. GenomeStudio software and the calling algorithm of Illumina were used to normalize the signal intensity. Finally, 700,078 SNVs were genotyped for each individual. Before genotype imputation, a series of strict quality controls were used to derive high-quality SNVs, with the following exclusion criteria: (1) samples with SNV genotyping rate less than 95%; (2) SNVs genotyped in less than 95% samples; (3) SNVs with a *P-*value of Hardy-Weinberg smaller than 1E-06; (4) SNVs with minor allele frequency (MAF) less than 5%.

Genotype imputation was performed against the China Metabolic Analytics Project (ChinaMAP, http://www.mbiobank.com), a large-scale and high-depth whole-genome sequencing dataset based on the Chinese population. This reference panel was constructed based on the ChinaMAP phase1 dataset, which includes 136,745,826 SNVs in 10,588 individuals. Our clean genotyped data were imputed by the ChinaMAP Imputation Server. After imputation, SNVs with the *R*^2^ smaller than 0.3 and/or SNVs with the *P*-value of Hardy–Weinberg smaller than 1 × 10^−06^ were excluded. At last, 4,933,061 high-quality SNVs of 1,776 CAD patients passed the quality assessment.

### Generation of polygenic risk score for all-cause death

GWAS summary statistics for 15 traits, including CAD, myocardial infarction, ischemic stroke, heart failure, angina, type 2 diabetes (T2D), body mass index (BMI), high-density lipoprotein cholesterol (HDLC), low-density lipoprotein cholesterol (LDLC), total cholesterol (CHOL), triglycerides (TG), systolic blood pressure (SBP), diastolic blood pressure (DBP), C-reaction protein (CRP), and platelet levels, were downloaded from Biobank Japan (Supplementary Table S1). The GWAS effect size were transferred to our data to construct PRS scores for our patients via a pruning and thresholding approach implemented in PRSice2 (22). Briefly, clumping was performed on our patients' genotypes to remove SNVs in linkage disequilibrium with the most significant SNVs (*r*^2^ > 0.1) within a 250 kb window. Subsequently, under each trait, different PRS models were constructed based on different SNVs, which were selected by incrementing *P*-value threshold from 5 × 10^−8^ to 0.5 in a few hundred steps. Thus, hundreds of PRS models were generated under each trait. Next, to identify the optimal PRS model per trait for predicting death, under each model, all PRS scores were used as independent variables for fitting a logistic regression with all-cause death. Among hundreds of regression results, the model with the maximum fitted R-square was defined as the optimal PRS model for that trait for predicting death.

### Construction of metaPRS

To construct the metaPRS, the optimal PRS models having significant association (*P* < 0.1) with all-cause death were identified by Cox regression, adjusting for age, sex, medication including proton pump inhibitors (PPI), angiotensin-converting enzyme inhibitors (ACEI), β-blockers (BB), and calcium channel blockers (CCB), and the first 10 principal components (PCs) of the genotypes. Next, Elastic-net Cox regression with 5-fold cross-validation was used to construct the metaPRS by combining all significant PRS models using the R package “glmnet”, which was proven effective in the presence of correlation between each PRS (23). A series of different metaPRS models were generated by tuning the parameter λ with different penalties, conferring different weights to each independent variable – in this case, the selected optimal PRS models. The performance of each metaPRS model was evaluated by the area under the operator curves (AUC), and the effect size of each optimal PRS model was determined when the AUC reached its maximum value among all penalties. At last, the metaPRS for death was determined from the model with the largest AUC value.

### Statistical analysis

In this study, the baseline characteristics of patients were presented as mean ± standard deviation (SD) for continuous variables and count (percent) for categorical variables. A two-tailed Students' *t*-test was used to identify the difference of continuous variables between patients with or without death, χ^2^ test was used to detect the difference between two groups with normally distributed data, and the Mann–Whitney *U*-test was used to identify the difference between two groups with non-normally distributed data. A *P*-value less than 0.05 was considered statistically significant. Pearson correlation analysis was used to identify the relationship between each optimal PRS. These patients were divided into three groups with low [≤quartile (Q) 1], intermediate (>Q1 and <Q3), and high risk of death (≥Q3) based on the quartiles of the calculated metaPRS for all individuals. The AUC values of all predictive models were calculated using the R package “timeROC”. Sensitivity and specificity of the prediction models were calculated based on the function of confusionMatrix implemented in the R package “InformationValue”.

## Results

### Baseline characteristics of the study population

The overall study design was illustrated in Figure 1. We utilized a prospective cohort design and recruited 1,776 patients with CAD. During a follow-up period of up to 11 years, 171 patients experienced all-cause death, and the death rate of patients in this study was illustrated in Supplementary Figure S1. Characteristics of the patients at enrollment, i.e., baseline, were displayed in Table 1. The patients were predominantly male (79.56%), both in the death group and the survival group. The mean age of patients at enrollment was significantly older in the death group than in the survival group (*P* = 5.37 × 10^−18^). In addition, patients in the death group were inclined for diabetes mellitus, heart failure, and arrhythmia, and their blood levels of aspartate aminotransferase, creatine kinase, creatine, and glucose were higher (*P* < 0.05, Table 1). Overall, baseline characteristics of these patients were consistent with the epidemiologic findings.

**Table 1:** Baseline characteristics of the participants in the CAD prognostic cohorts.

Characteristics	Death (*N* = 171)	Survival (*N* = 1,605)	*P*-value
	*N* (%) or mean ± SD	*N*(%) or mean ± SD	
Demographic data			
Age (year)	69 ± 9	62 ± 10	<0.0001
Male	142 (83.04)	1,271 (79.09)	0.28
Medical history			
Diabetes mellitus	72 (42.11)	420 (26.22)	<0.0001
Hypertension	108 (63.16)	934 (58.27)	0.25
Heart failure	45 (26.32)	194 (12.10)	<0.0001
Arrhythmia	28 (16.37)	48 (7.08)	0.0002
Biomedical measurements			
ALT, U/L	29 ± 17	27 ± 14	0.13
AST, U/L	35 ± 66	26 ± 11	<0.0001
LDLC, mmol/L	2.61 ± 0.97	2.67 ± 0.99	0.45
HDLC, mmol/L	0.97 ± 0.24	0.99 ± 0.24	0.31
Triglycerides, mmol/L	1.60 ± 1.20	1.64 ± 1.12	0.69
ApoA, g/L	1.02 ± 0.28	1.07 ± 0.26	0.01
TC, mmol/L	4.25 ± 1.14	4.34 ± 1.25	0.38
LPA, mg/dl	333.51 ± 350.81	290.69 ± 315.30	0.13
CK, U/L	150.10 ± 591.76	111.16 ± 106.83	0.02
CKMB, U/L	8.5 ± 8.1	8.0 ± 6.1	0.39
Creatinine, µmol/L	102 ± 38	86 ± 39	<.0001
Glucose, mmol/L	7.11 ± 2.68	6.54 ± 2.61	0.007
Medication			
PPI	104 (60.82)	863 (53.77)	0.09
ACEI	105 (61.40)	949 (59.13)	0.62
BB	151 (88.30)	1,416 (88.22)	0.99
CCB	70 (40.94)	429 (26.73)	0.0001

ALT, alanine aminotransferase; AST, aspartate aminotransferase; LDLC, low-density lipoprotein cholesterol; HDLC, high-density lipoprotein cholesterol; ApoA, apolipoprotein A; TC, total cholesterol; LPA, Lipoprotein (a); CK, creatine kinase; CKMB, creatine kinase MB; PPI, proton pump inhibitors; ACEI, angiotensin-converting enzyme inhibitors; BB, β-blockers; CCB, calcium channel blockers.

### Association between death and polygenic risk scores of CAD and its risk factors

**Figure 2:**
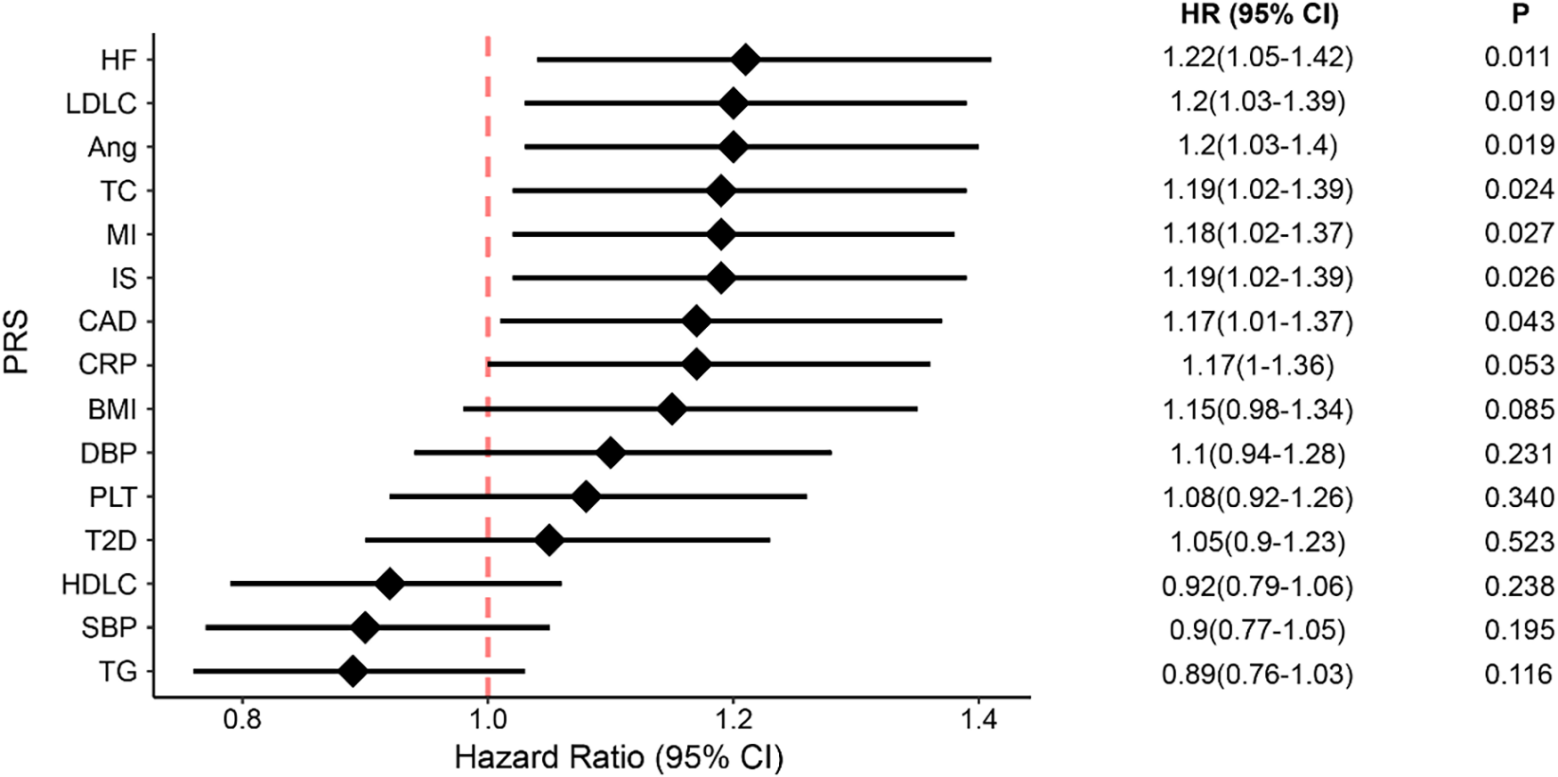
The associations between all-cause death and genetic susceptability of CAD and its 14 risk factors, presented in PRS scores. The diamonds represent the coefficients and the error bars represent the 95% confidential interval. HF, heart failure; Ang, angina; MI, myocardial infarction; IS, ischemic stroke; CRP, C-reaction protein; CAD, coronary artery disease; BMI, body mass index; DBP, diastolic blood pressure; SBP, systolic blood pressure; PLT, platelet; T2D, type 2 diabetes; LDLC, low-density lipoprotein cholesterol; HDLC, high-density lipoprotein cholesterol; TC, total cholesterol; TG, triglycerides; CI, confidence interval.

We evaluated whether genetic susceptibility to CAD and its risk factors could predict all-cause death in patients with prior CAD. As such, we chose to study CAD and its 14 risk factors, namely myocardial infarction, ischemic stroke, heart failure, angina, T2D, BMI, HDLC, LDLC, CHOL, TG, SBP, DBP, CRP, and platelet levels. For each trait, we transferred the GWAS effect sizes, i.e., the beta values, learned in the East Asians in Biobank Japan, to compute PRS scores for our patients. For each trait, we used different significance thresholds (*P*-values ranged from 5 × 10^−08^ to 0.5) to select different risk alleles and computed the PRS, thus deriving a series of PRS scores. We then performed logistic regression, treating each PRS score as an independent variable and adjusting for covariates including sex, age, medications, and the first 10 principal components (PC) of the genotypes, for identifying which PRS model of that particular trait was best associated with all-cause death in our cohort. The optimal PRS was defined as having the biggest effect size in the association tests. As such, we derived 15 optimal PRS models and their scores for each patient in our cohort (Supplementary Table S1). We discovered that the PRS scores of seven traits, i.e., CAD, IS, MI, HF, Angina, TC, and LDLC, showed significant association with all-cause death (*P* < 0.05, Figure 2), and CRP and BMI showed weak significance with death (*P* < 0.1, Figure 2). In total, nine PRS models displayed significant association with all-cause death with the direction of effect consistent with the epidemiologic findings.

**Figure 3:**
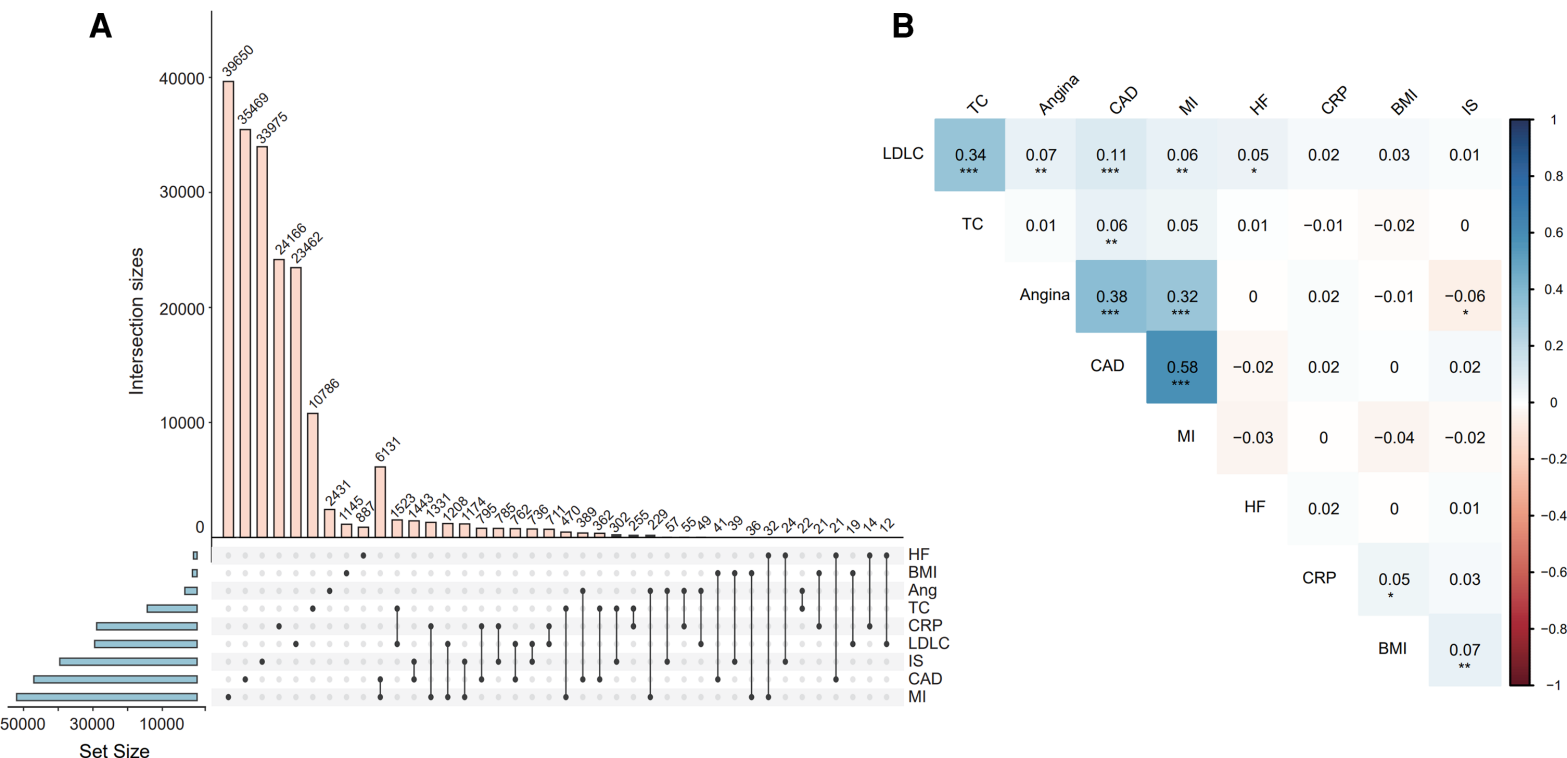
The intersection of SNVs for constructing each PRS model (**A**) and the correlation between the PRS models (**B**). Correlation coefficients and *P*-values were estimated from the Pearson correlation test for each pair of PRSs. *Indicates *P* < 0.05, ** indicates *P* < 0.01, *** indicates *P* < 0.001. Abbreviations refer to Figure 2.

Next, we explored the overlap of variants used to compose the nine PRS models and their pairwise correlations. The number of variants in each model ranged from 1,026 to 51,982 (Figure 3A), exhibiting varying degrees of overlap. PRS of CAD overlapped the most with myocardial infarction, resulting in the strongest positive correlation between the two models (*r* = 0.58, Pearson correlation *P* < 0.001). Furthermore, the PRS of CAD significantly correlated with angina, LDLC, and TC (Figure 3B), and PRS of LDLC significantly correlated with all ascertained cardiovascular diseases, namely angina, CAD, MI, and HF.

### Predicting death risk by a meta polygenic risk score

Given that the nine PRS models correlated with each other to different degrees, we adopted the elastic-net Cox regression approach with 5-fold cross-validation to integrate them all into a metaPRS model, which consisted of 193,312 unique SNVs. As expected, the metaPRS had the strongest association with all-cause death (HR: 1.85; 95% CI: 1.50–2.28) compared to the single PRS models (Figure 2).

**Figure 4:**
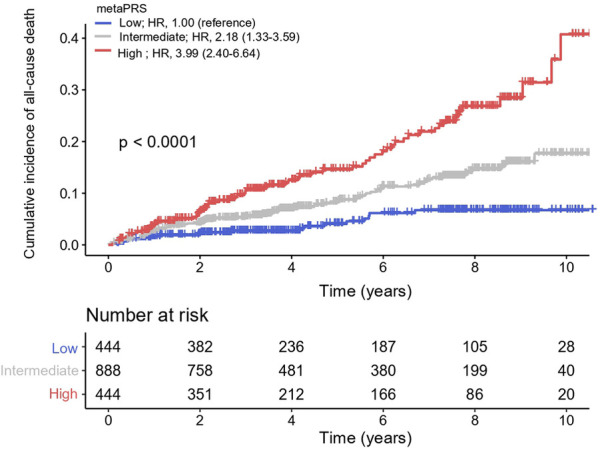
Cumulative incidence of all-cause death across three metaPRS categories. Cox proportional hazards model was used to estimate the hazard ratios (95% confidence intervals). Polygenic risk categories: low (≤Q1), intermediate (>Q1 and <Q3), and high risk (≥Q3) according to the quantiles of metaPRS. HR, hazard ratios; CI, confidence interval.

For assessing its ability in risk stratification, we divided our patients into three groups by the quartiles of metaPRS scores, with the scores less than the first quartile (≤Q1) and greater than the third quartile (≥Q3) as the low and the high risk groups, respectively, and the scores between first and third quartiles (>Q1 and <Q3) as the intermediate risk group. We found that the cumulative incidence of all-cause death was significantly greater in CAD patients in the third quartile than in the first quartile, with the HR of 3.99 (95% CI: 2.40–6.64) per SD increment (*P* = 9.10 × 10^−8^), and the cumulative incidence of all-cause death in CAD patients with intermediate metaPRS was also significantly greater than in the first quartile, with the HR of 2.18 (95% CI: 1.33–3.59) per SD increment (*P* = 2.10 × 10^−3^) (Figure 4).

### Predicting death risk by polygenic and clinical risk scores

Other than genetic susceptibility, environmental factors and lifestyles also contribute to the incidence and progression of cardiovascular diseases. For example, age is a known strong predictor. Therefore, we explored whether combining metaPRS and clinical risk factors could improve the prediction power for all-cause death in CAD. First, we evaluated single risk factors and found that age had a good predictability with an AUC of 0.69 (Supplementary Table S2), followed by smoking (AUC = 0.58) and sex (AUC = 0.54). As a reference, the metaPRS model achieved an AUC of 0.63. Next, we combined the three clinical factors and achieved an AUC of 0.74. Combining metaPRS with the three clinical risk factors resulted in a further increase of AUC to 0.76.

**Figure 5:**
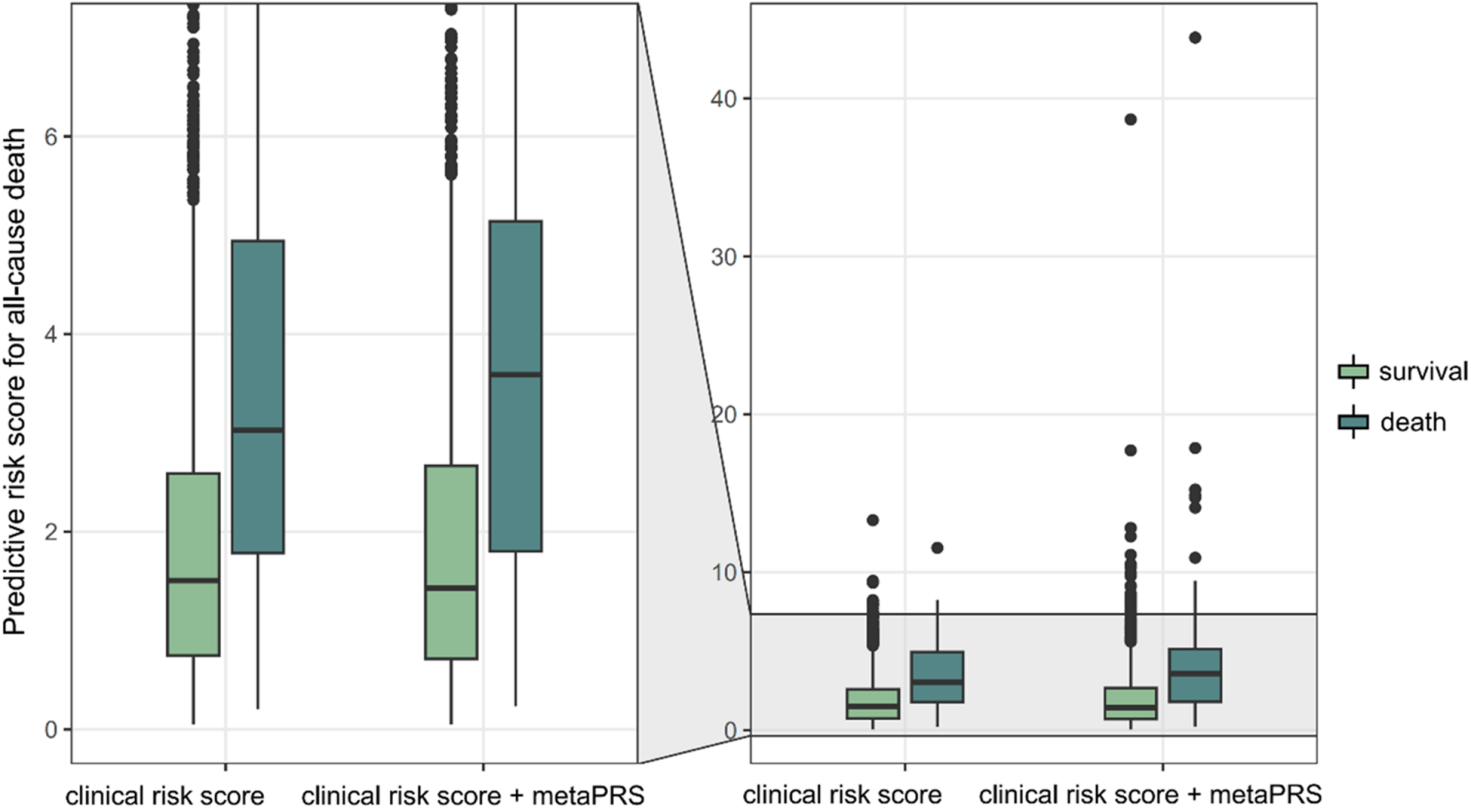
Predictive risk of all-cause death. Two models, the clinical risk model comprising of age, sex and smoking status, and the combined model comprising of the three clinical risk score and metaPRS, are compared.

Close examination of the prediction scores revealed that combining metaPRS with the clinical risk factors, which we termed as the combined model, significantly improved prediction than using the clinical risk factors alone (Figure 5). While no difference was observed for the survival group between the combined model and the clinical risk model, an increase in prediction scores was observed in the death group using the combined model. Furthermore, we observed a 4% increase in sensitivity after adding metaPRS to the clinical risk model, while the specificity remained unchanged (Table 2). Taken together, these results indicate that combining genetic and clinical risk factors is most advantageous in identifying patients at elevated risk of death, which is critical to enhancing secondary preventions in CAD patients.

**Table 2:** Sensitivity analysis for metaPRS, clinical model and the combination of clinical and metaPRS model.

	Reference	metaPRS		Clinical model		Clinical + metaPRS model	
Predictive		Survival	Death	Survival	Death	Survival	Death
Survival		954	70	1,146	66	1,136	60
		(TN)	(FN)	(TN)	(FN)	(TN)	(FN)
Death		651	101	459	105	469	111
		(FP)	(TP)	(FP)	(TP)	(FP)	(TP)
Totals		1,605	171	1,605	171	1,605	171
Sensitivity			59%		61%		65%
Specificity		59%		71%		71%	
Cutoff value		1.095		2.4			

TN, true negative; TP, true positive; FN, false negative; FP, false positive.

## Discussion

In this study, we evaluated whether genetic susceptibility to CAD and its risk factors could predict future death in CAD patients. Our results showed that the PRSs for CAD and its eight risk factors were significantly associated with death. Meanwhile, we developed a metaPRS model for predicting death, which could well stratify CAD patients of low, intermediate, and high risks of death. In addition, we found that combining metaPRS with clinical risk factors moderately increased the prediction power compared with using clinical risk factors alone.

Previous large-scale GWAS studies were predominantly conducted in European ancestry populations and focused on the incidence of CAD (15, 16), leading to an insufficient discovery both in East Asian ancestry and adverse outcomes of CAD. The large-scale meta-GWAS analysis of CAD and its risk factors conducted by Biobank Japan (9, 10) allowed us to apply their learning in the East Asian population to study the impact of genetics on CAD progression in our Chinese patients. The good stratification of patients at low, intermediate, and high risk of death by our proposed metaPRS model supports the notion that genetics not only contributes to the incidence but also progression of CAD. Our metaPRS model provides a valid quantification for such genetic contribution.

The prognosis of CAD is a complex conversation driven by both genetic and nongenetic factors, such as environmental exposure and lifestyles (24), presenting a challenge in predicting future adverse outcomes. In this study, we found that PRS of CAD had a positive association with all-cause death in CAD patients, after adjusting for traditional risk factors and medication usage. This was consistent with previous studies, such as Odyssey Outcome Trial and Fourier Trial that CAD patients of higher PRS would benefit better from the medical treatments from death and major adverse cardiovascular events (25–28). Meanwhile, we noticed that all PRS scores for severe and even fatal events such as myocardial infarction, heart failure, and ischemic stroke harbored greater associations with death in the CAD patients than the PRS of CAD itself, suggesting that genetic susceptibility to CAD reflected broader spectrum of disease conditions but was less effective in predicting adverse outcomes, whereas genetic susceptibility to more extreme conditions confer greater risk to the patients. Thus, our study highlights the importance of considering overall genetic profiles, particularly those associated with fatal conditions, when developing tools for the secondary prevention of CAD.

In addition to predicting the incidence of CAD (18), our study revealed that age was also the biggest contributor to predicting future death in CAD patients. Although the addition of metaPRS to the clinical risk factors achieved only a modest improvement in the AUC value, the median of the predictive risk scores had an obvious elevation in the death group, which in turn resulted in a 4% increase in sensitivity. Studies suggest that restoring a healthy lifestyle and increasing physical activity may reduce the risk of cardiovascular death and all-cause death, even in patients with the highest genetic risks (24, 29, 30). Thus, predicting a long-term risk solely based on age may lead to an inaccurate estimation. Genetic risk supplements the picture by providing an independent component, which can be detected early in life and has demonstrated promises in predicting future outcomes (31, 32). Our results showed that combining genetic risk with clinical risk factors achieved the best prediction power. Indeed, the combined model increased the risk scores for patients in the death group while maintaining the scores at similar levels for patients in the survival group, suggesting the genetic risk harbored specificity and did not broadly inflate the risk scores. Such specificity confers the combined model the capability to identify patients at higher risk for adverse outcomes.

We highlight the strengths and limitations of this study. For the strengths, first, we recruited a prospective CAD cohort with detailed baseline information and up to 11 years of follow-up on all-cause death, which allowed us to explore comprehensively the genetic profile of CAD and its risk factors and their ability to predict a 10-year death risk in the CAD patients. Second, this study filled a gap in utilizing PRS for secondary prevention of CAD in East Asian populations. However, our study also has limitations. First, the cohort enrolled in this study has a small sample size relative to biobank studies such as UK Biobank. Second, this study did not include an external validation cohort to verify the performance of metaPRS, due to the lack of CAD patient cohorts with long-term follow-up. Future studies are warranted for validation.

## Conclusions

In summary, our study developed a polygenic risk score with good promising in facilitating the identification of patients at higher risk of developing adverse outcomes. Incorporating polygenic risk scores into clinical care may provide a valuable guidance on risk stratification for early identification of patients who would benefit from intensive lifestyle changes and drug treatment.

## Data Availability

The datasets presented in this study can be found in online repositories. The names of the repository/repositories and accession number(s) can be found below: EMBL-EBI [Project: PRJEB42554; Analyses: ERZ1714343].
