# Supplementary material for: Polygenic risk score predicts all-cause death in East Asian patients with prior coronary artery disease
Source: Front Cardiovasc Med. 2024 Feb 13;11:1296415. doi: 10.3389/fcvm.2024.1296415 (PMC10896892; doi:10.3389/fcvm.2024.1296415)
Supplement: Supplementary file 1 [file Table1.docx]

**Supplementary materials**

**List of contents**

Supplementary Figure 1. The survival probability of patients in this study.

Supplementary Table S1. The sources of genome-wide association studies for the selected diseases and traits

Supplementary Table S2. The area under the curve (AUC) value of metaPRS and clinical risk factors

**
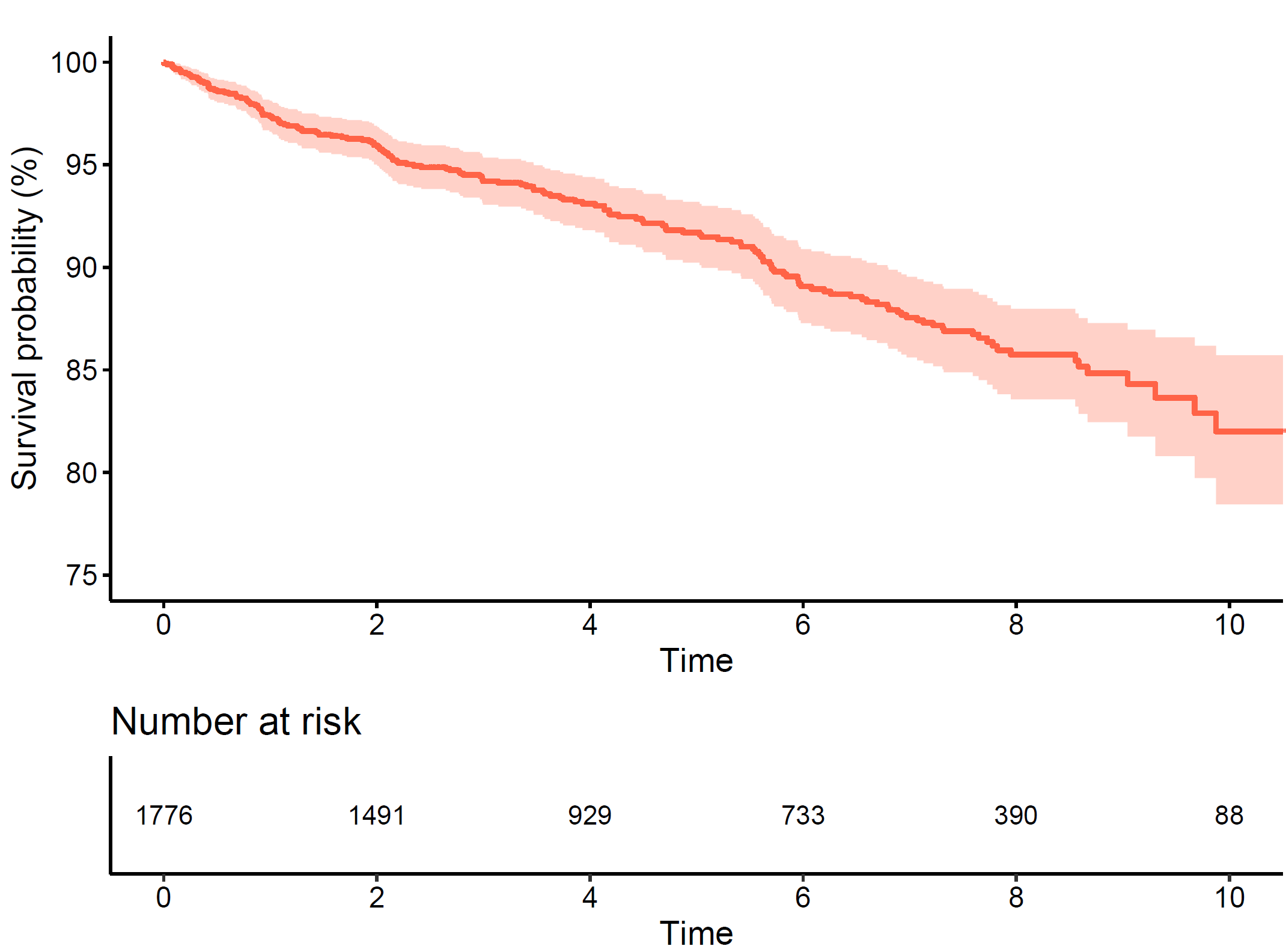
**

**Supplementary Figure 1. The survival probability of patients in this study.**

**Supplementary Table S1. The sources of genome-wide association studies for the selected diseases and traits.**

| **Project** | **PMID** | **Traits** | **Ncase** | **Ncontrol** | **Ntotal** |
| --- | --- | --- | --- | --- | --- |
| BBJ | 29403010 | CAD | 29319 | 183134 | 212453 |
| BBJ | 29403010 | IS | 17671 | 192383 | 210054 |
| BBJ | 34594039 | MI | 14992 | 146214 | 161206 |
| BBJ | 34594039 | T2D | 45383 | 132032 |  |
| GBMI | / | HF | 12665 | 245263 | 257928 |
| BBJ | 34594039 | Angina | 14007 | 145158 | 159165 |
| BBJ | 34594039 | BMI | / | / | 163835 |
| BBJ | 34594039 | Platelet | / | / | 148623 |
| BBJ | 34594039 | TC | / | / | 135808 |
| BBJ | 34594039 | HDLC | / | / | 74970 |
| BBJ | 34594039 | LDLC | / | / | 72866 |
| BBJ | 34594039 | TG | / | / | 111667 |
| BBJ | 34594039 | CRP | / | / | 83025 |
| BBJ | 34594039 | SBP | / | / | 145505 |
| BBJ | 34594039 | DBP | / | / | 145515 |

BBJ: Biobank Japan Project (BBJ); GBMI: Global Biobank Meta-analysis Initiative. CAD: coronary artery disease; IS: ischemic stroke; MI: myocardial infraction; T2D: type 2 diabetes; HF: heart failure; BMI: body mass index; TC: total cholesterol; HDLC: high-density lipoprotein cholesterol; LDLC: low-density lipoprotein cholesterol; TG: triglycerides; CRP: C-reaction protein; SBP: systolic blood pressure; DBP: diastolic blood pressure.

**Supplementary Table S2. The area under the curve (AUC) value of metaPRS and clinical risk factors**

| **Predictive models** | **AUC** |
| --- | --- |
| Age | 0.69 |
| Sex | 0.54 |
| Smoking | 0.58 |
| metaPRS | 0.63 |
| Age + sex | 0.7 |
| Age + SeX + Smoking | 0.74 |
| Age + Sex + Smoking + metaPRS | 0.76 |
